# Comprehensive Analysis of *HMCN1* Somatic Mutation in Clear Cell Renal Cell Carcinoma

**DOI:** 10.3390/genes13071282

**Published:** 2022-07-20

**Authors:** Ziqi Gong, Xiaowen Wu, Qian Guo, Haizhen Du, Fenghao Zhang, Yan Kong

**Affiliations:** Key Laboratory of Carcinogenesis and Translational Research (Ministry of Education/Beijing), Department of Renal Cancer and Melanoma, Peking University Cancer Hospital and Institute, Beijing 100142, China; gongziqi1997@foxmail.com (Z.G.); wuxiaowen2013@bjmu.edu.cn (X.W.); liziqian15@163.com (Q.G.); duhaizhen2018@163.com (H.D.); zhangfenghao6949@163.com (F.Z.)

**Keywords:** bioinformatics, clear cell renal cell carcinoma, *HMCN1*, immunity, metabolism, mutation, prognosis

## Abstract

Background: Renal cell carcinoma (RCC) is a common malignancy of the genitourinary system and clear cell renal cell carcinoma (ccRCC) is the most representative subtype. The morbidity and mortality of ccRCC have gradually risen during recent years; however, the pathogenesis and potential biomarkers remain unclear. The purpose of our study was to find out prognostic genes correlated with somatic mutation and the underlying mechanisms of *HMCN1* mutation in ccRCC. Methods: Somatic mutation data of two ccRCC cohorts were acquired from TCGA and cBioPortal. Genes frequently mutated in both datasets were extracted, from which tumor mutation burden and survival analysis revealed three prognostic genes. Further comprehensive analysis of *HMCN1* mutation was carried out to identify differentially expressed genes and apply functional annotations. The correlation of *HMCN1* mutation and tumor immunity was also evaluated. Results: *HMCN1*, *SYNE1*, and *BAP1* mutations were associated with both tumor mutation burden and clinical prognosis in ccRCC. Gene enrichment analysis suggested the effects of *HMCN1* mutation on biological processes and pathways linked to energy metabolism. *HMCN1* mutation was also correlated with anti-tumor immunity. There were several limitations in the sample size and cohort availability of the present computational study. Conclusions: The present results inferred that *HMCN1* mutation might have an important clinical significance for ccRCC patients by regulating metabolism and the immune microenvironment.

## 1. Introduction

Renal cell carcinoma (RCC) is a common malignant solid tumor of the urinary system with a morbidity of approximately 400,000 cases worldwide [1,2]. RCC are heterogeneous tumors with multiple histological subtypes [3]. The most representative one is clear cell renal cell carcinoma (ccRCC), which makes up approximately 70% of all cancers of the kidney [4].

Since there are no obvious symptoms and reliable diagnostic biomarkers at the beginning, 25–30% of RCC patients have already developed spread and metastasis by the time of diagnosis [5]. In the early stages, radical nephrectomy is the most effective treatment [6], but recurrence or metastasis occurs in 30% of patients after surgery [7,8]. Because of its intrinsic resistance, RCC is insensitive to both chemotherapy and radiotherapy, which makes the administration of advanced RCC a challenge [2,9]. Immune checkpoint inhibitor therapy has been widely applied in many types of tumors in the past few years, including ccRCC [10], but the therapeutic effectiveness is still controversial [2]. Although there have been significant improvements in the diagnosis and management of ccRCC, the underlying pathological mechanisms and prognosis-related biomarkers remain to be explored.

Somatic gene mutation is considered to be critical in disease programs, and different gene mutation statuses may affect tumor development, clinical outcomes, and treatment strategies. Tumor mutation burden (TMB) is a biomarker of the somatic mutation status in tumors, which serves as a valid predictor for the responses to immunotherapy [11,12]. Recent studies have also shown that at the cellular level, the infiltration of different subsets of immune cells was involved in antitumor responses and may also be associated with gene mutation status [13,14].

We performed bioinformatic analysis of somatic mutations combined with TMB and clinical prognosis in the present study, identifying *HMCN1* (Hemicentin 1) mutation as a critical event in ccRCC progression. HMCN1 belongs to the family of extracellular matrix (ECM) proteins [15]. Previous studies suggest that *HMCN1* is primarily associated with age-related macular degeneration (AMD) [16]. HMCN1s usually localize in the extracellular stroma, so they may also promote tumor invasion or metastasis [17]. Mutations and the abnormal expression of *HMCN1* have been found in a variety of tumors, but its function and molecular mechanisms in ccRCC are still unclear. To this end, we also carried out comprehensive analyses to find out the novel pathways in *HMCN1*-mutant samples and elucidated that the potential mechanisms of *HMCN1* mutation were related to cellular metabolism and anti-tumor immunity in ccRCC development.

## 2. Materials and Methods

### 2.1. Data Acquisition

Single nucleotide variation (SNV) data of 537 ccRCC patients (mainly from western countries) was downloaded from TCGA [18]. We also downloaded the corresponding clinical information and transcriptome profile of TCGA cohort. SNV data of 106 Japanese ccRCC patients from Tokyo were extracted from UTokyo cohort [19], which was acquired from cBioPortal [20].

### 2.2. Genome-Wide Mutation Profiling

We analyzed and visualized the somatic mutation characteristics in each sample with Perl software and R package “GenVisR” [21]. The frequency of various variant classifications and distribution of different types of variant genes were visualized by the waterfall plot. We intersected the top 30 genes ranked by mutation rate in both groups.

### 2.3. TMB Calculation and Survival Analysis

TMB was defined as the mutation rate per million bases [22], which was assessed by dividing non-synonymous mutation numbers by the exon length. Non-synonymous mutations contain all missense, insertion/deletion, and frameshift variants [23,24]. In present study, TBM in each TCGA sample was calculated by Perl. The correlation of TMB score and gene mutation status was analyzed by R package “ggpubr”. Then, Kaplan–Meier survival analysis and log-rank test was performed for evaluation with R packages “survminer” and “survival”. We also built a Cox proportional hazards model consisting of all clinical pathological characteristics via univariate and multivariate Cox regression. 

### 2.4. Differentially Expressed Genes (DEGs) Analysis

We employed the DEGs analysis to identify DEGs between *HMCN1*-mutant and wild-type samples with a cutoff of |Log FC| > 1 and FDR < 0.05. The resulting volcano map and heatmap were plotted by R package “edge R” and “pheatmap” [25]. 

### 2.5. Functional Annotation of DEGs

Gene enrichment analysis was processed by GO and KEGG and visualized by R package “clusterProfiler”, “enrichplot”, and “ggplot2” [26]. Gene set enrichment analysis (GSEA) was also performed by GSEA software (v4.2.2) to analyze gene expression profile at the gene-set level [27]. Pathways with a *p* value < 0.05 were considered statistically significant. Gene sets “c2.cp.kegg.v7.5.1.symbols.gmt” and “h.all.v7.5.1.symbols.gmt” applied to GSEA were obtained from Molecular Signature Database [28]. 

### 2.6. Protein–Protein Interaction (PPI) and Submodule Analysis

To construct a PPI network, we used the online tool STRING [29]. The network was screened with combined score more than 0.4 and visualized with Cytoscape 3.9.1 [30]. Submodule analysis was carried out by MCODE [31] to identify hub modules that may perform independent functions. We selected the most highly connected modules with a degree cutoff = 2, node score cutoff = 0.2, k-core = 2, and max depth = 100. Functional annotations were performed subsequently for screened hub subnetworks.

### 2.7. Estimation of Immune Cell Infiltrating

We evaluated the infiltrating levels of 22 types immune-infiltrating cells according to CIBERSORT [32]. After calculation, the composition of different immune cells was demonstrated. Then R package “limma” was used to perform Wilcoxon rank-sum test in patients with different *HMCN1* mutation statuses [33]. The result was visualized in the violin plot by R package “vioplot” [34]. We also compared the expression of immune checkpoint genes between *HMCN1*-mutant and wild type samples by R package “limma”.

### 2.8. Statistical Analysis

R software (version 4.1.2) was applied for all statistical analyses. The correlation of gene mutation and TMB score was investigated by Mann–Whitney U test. Survival curves were generated by Kaplan–Meier survival analysis, and log-rank test was applied for evaluation. Univariate and multivariate Cox regression models were constructed for each clinical risk factor. A two-tailed *p* value < 0.05 was considered statistically significant in all comparisons.

## 3. Results

### 3.1. Somatic Mutation Landscape of ccRCC

**Figure 1:**
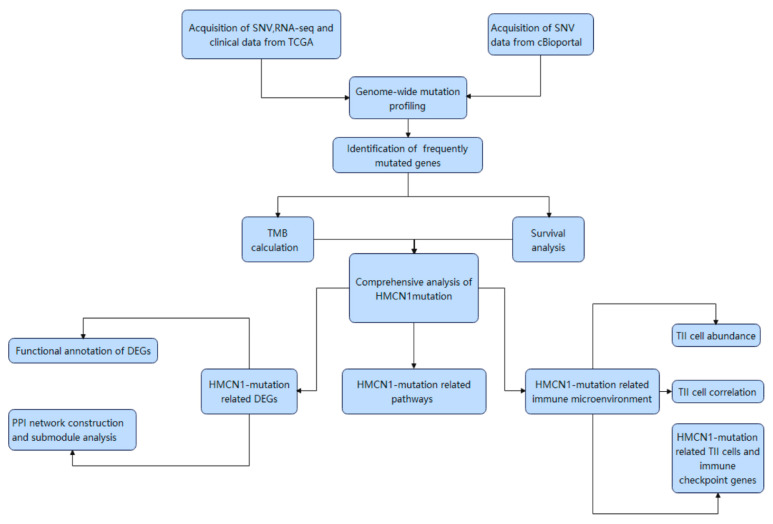
Workflow chart of the analysis process.

**Figure 2:**
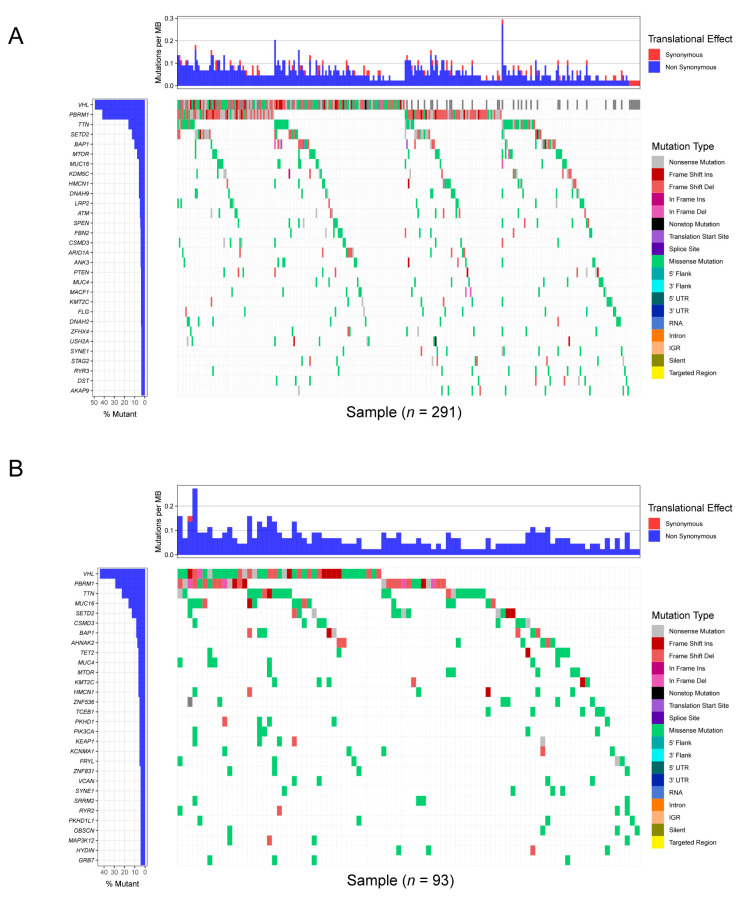
Profile of frequently mutated genes in KIRC. (**A**) Waterfall plot illustrating the top 30 genes in TCGA cohort. (**B**) Waterfall plot demonstrating the top 30 genes in UTokyo cohort.

**Figure 3:**
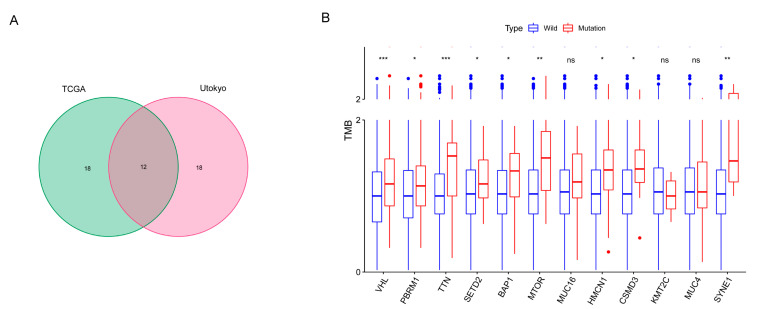
TMB-related gene mutations. (**A**) Venn diagram figures out 12 frequently mutated genes included in both two cohorts. (**B**) Boxplot reveals the correlation of gene mutation and TMB. *: *p* < 0.05; **: *p* < 0.01; ***: *p* < 0.001; ns: *p* > 0.05.

Figure 1 illustrates the workflow chart of our research. We first downloaded the required data for analysis from TCGA and cBioPortal and evaluated the variants of each sample in two cohorts. Detailed mutation information of the top 30 mutated genes is illustrated in Figure 2A,B, and different mutation types are distinguished by different color annotations. The top 10 mutated genes in TCGA cohort were *VHL*, *PBRM1*, *TTN*, *SETD2*, *BAP1*, *MTOR*, *MUC16*, *KDM5C*, *HMCN1*, and *DNAH9*. The top 30 mutated genes were figured out in Japanese patients from the UTokyo cohort as well. The most frequently mutated gene was *VHL*, followed by *PBRM1*, *TTN*, *MUC16*, *SETD2*, *CSMD3*, *BAP1*, *AHNAK2*, *TET2*, and *MUC4*. The Venn diagram in Figure 3A indicates that there are 12 genes carrying a relatively high mutation rate in both two cohorts.

### 3.2. Gene Mutations Related to TMB and Prognosis

In order to sort out the hub genes that probably serve a critical role in ccRCC, we first calculated the TMB values in all patients. Combining the mutation data with the TMB expression profile, we revealed that the TMB values of ccRCC patients were significantly associated with several gene mutations, including *VHL*, *PBRM1*, *TTN*, *SETD2*, *BAP1*, *MTOR*, *HMCN1*, *CSMD3*, and *SYNE1* (Figure 3B).

**Figure 4:**
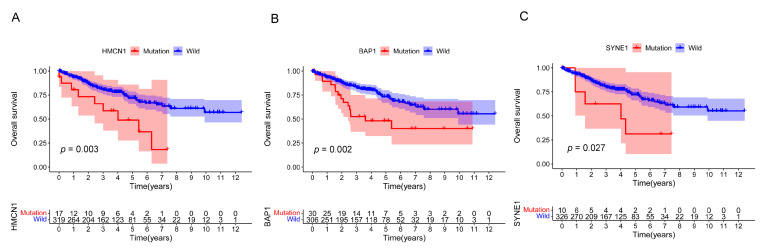
Gene mutations associated with prognosis. Kaplan–Meier survival curves of three gene mutations significantly correlated with clinical prognosis. (**A**): *HMCN1* mutation is associated with poorer prognosis; (**B**): *BAP1* mutation is associated with poorer prognosis; (**C**): *SYNE1* mutation is associated with poorer prognosis.

Then, we separated the patients in TCGA cohort into wild and mutant types depending on the gene mutation status and survival analysis was performed in combination with patients’ survival data. Kaplan–Meier survival curves and log-rank tests were conducted to figure out prognosis-related mutations. Our results demonstrated that among the 12 mutated genes, only 3 gene mutations were associated with the prognosis of ccRCC patients significantly, including *HMCN1*, *BAP1*, and *SYNE1*, which were also TMB-related genes (Figure 4A–C). The biological role and molecular mechanisms of *BAP1* and *SYNE1* mutations in ccRCC have been previously reported [35,36,37]. In this work, we further carried out an integrated analysis of *HMCN1* mutation.

**Figure 5:**
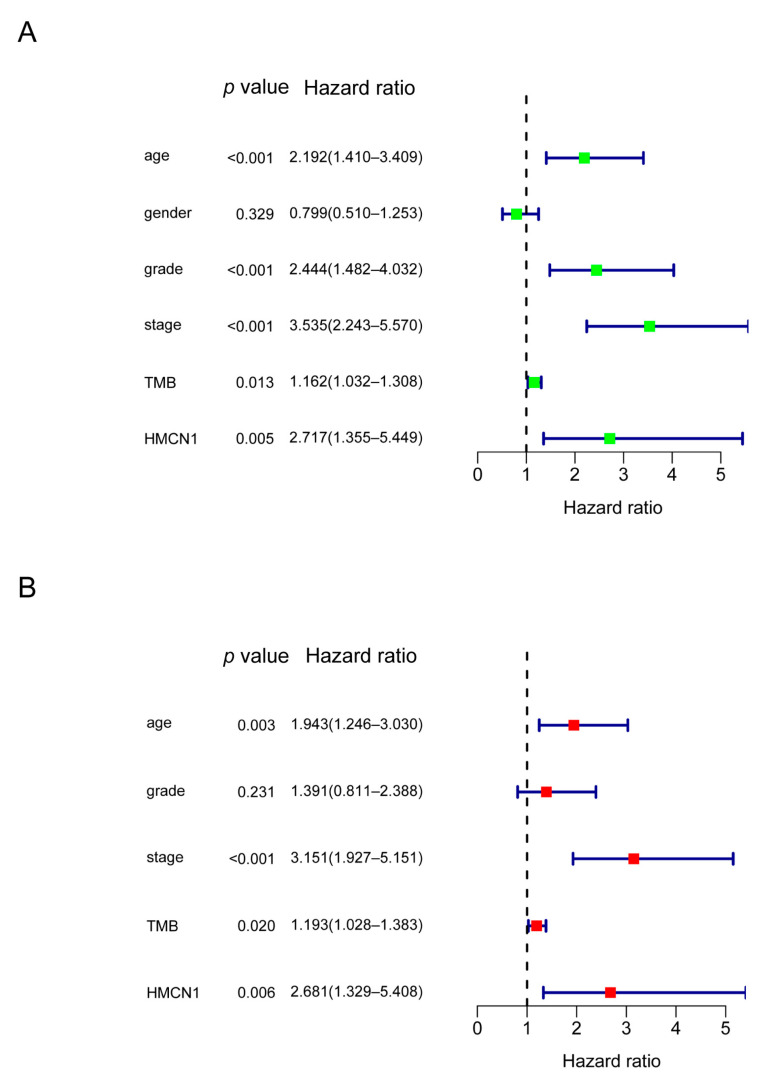
Univariate (**A**) and multivariate (**B**) Cox regression model of KIRC.

As was illustrated in Figure 4A, *HMCN1* mutation was significantly associated with poorer clinical outcome. To determine if *HMCN1* mutation was an independent predictive biomarker, further univariate and multivariate Cox regressions were employed. After the screening of a multivariate model, *HMCN1* mutation remained significantly associated with overall survival under correction for clinical characteristics and TMB score. In addition, age, stage, and TMB score were also considered as important prognosis-related biomarkers. Older age, a more advanced stage, and a higher TMB value were significantly associated with a poorer clinical prognosis (Figure 5A,B).

### 3.3. Identification of DEGs

**Figure 6:**
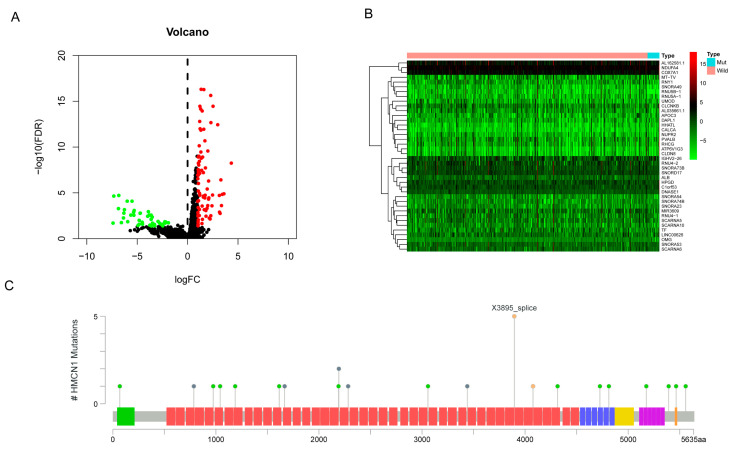
Differentially expressed genes analysis. (**A**) Volcano map of DEGs. Red: significantly upregulated genes; Green: significantly down-regulated genes. (**B**) Heatmap of DEGs. (**C**) *HMCN1* mutation types and sites in KIRC. Green: von Willebrand factor type A domain; Red: Immunoglobulin I-set domain; Blue: Thrombospondin type 1 domain; Yellow: G2F domain; Purple: Calcium-binding EGF domain; Orange: Complement Clr-like EGF-like.

We selected DEGs to further explore potential pathways by which *HMCN1* mutation impacts ccRCC development. The differentially expressed mRNAs in *HMCN1*-mutant and wild-type samples were calculated with a criteria of log fold change >1.0 or <−1.0 and FDR < 0.05. Overall, we identified 134 DEGs, with 88 being upregulated in *HMCN1*-mutant samples and 46 being downregulated. The results were illustrated by a volcano map and heatmap in Figure 6A,B. Moreover, the lollipop chart downloaded from cBioPortal demonstrated that the mutation types of *HMCN1* included splice, in-frame, and missense mutations across the entire gene (Figure 6C).

### 3.4. Functional Annotations of DEGs

**Figure 7:**
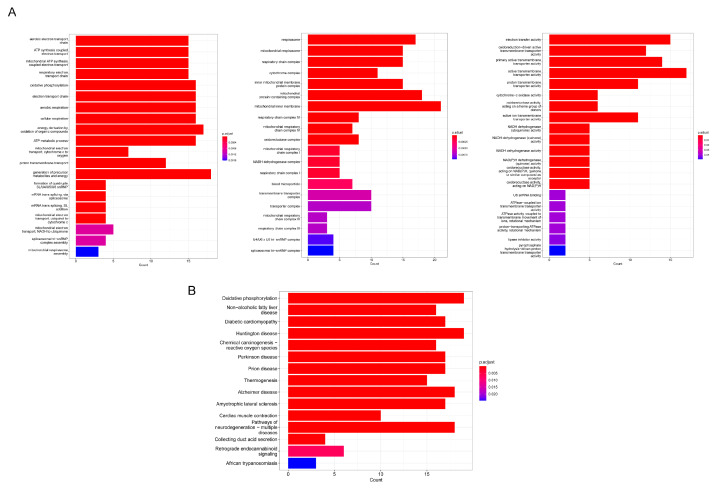
GO and KEGG analysis. (**A**) Bar graph of gene ontology functional annotations. (**B**) Bar graph of KEGG pathway enrichment analysis.

Gene enrichment analysis was performed after identifying DGEs. The top 20 GO and KEGG terms were illustrated in Figure 7A,B. GO analysis indicated that the main biological processes of genes differentially expressed in patients with *HMCN1* mutation were enriched in the aerobic electron transport chain, ATP synthesis coupled electron transport and mitochondrial ATP synthesis coupled electron transport. The main pathways enriched were oxidative phosphorylation, non−alcoholic fatty liver disease, and diabetic cardiomyopathy.

**Figure 8:**
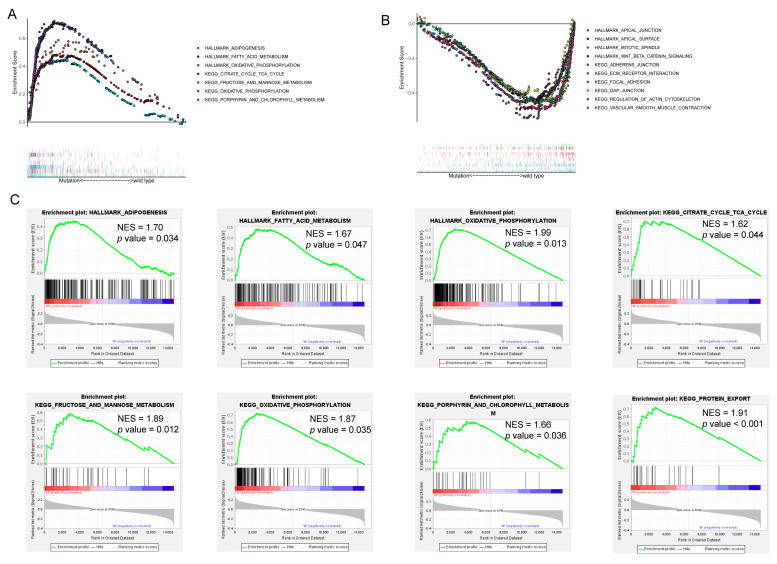
*HMCN1* mutation-related pathways. (**A**) Multigene enrichment plot shows gene sets enriched in *HMCN1*-mutant cases. (**B**) Multigene enrichment plot shows gene sets enriched in wild-type cases. (**C**) Several enrichment plots displaying a series of metabolism-related pathways.

GSEA further confirmed that *HMCN1* mutation may lead to alterations in key pathways related to energy metabolism. Figure 8A,C exhibited the hallmarks and pathways that considerably enriched in *HMCN1* mutant samples, containing adipogenesis, oxidative phosphorylation, fatty acid metabolism, the TCA cycle, fructose and mannose metabolism, and porphyrin and chlorophyll metabolism. Pathways including apical junction, apical surface, mitotic spindle, adherens junction, focal junction, gap junction, and regulation of the actin cytoskeleton were significantly enriched in wild-type samples. The majority of these were closely associated with cell polarity, the cytoskeleton, and cell junctions (Figure 8B).

### 3.5. PPI Network Establishment, Hub Genes, and Submodules Screening

**Figure 9:**
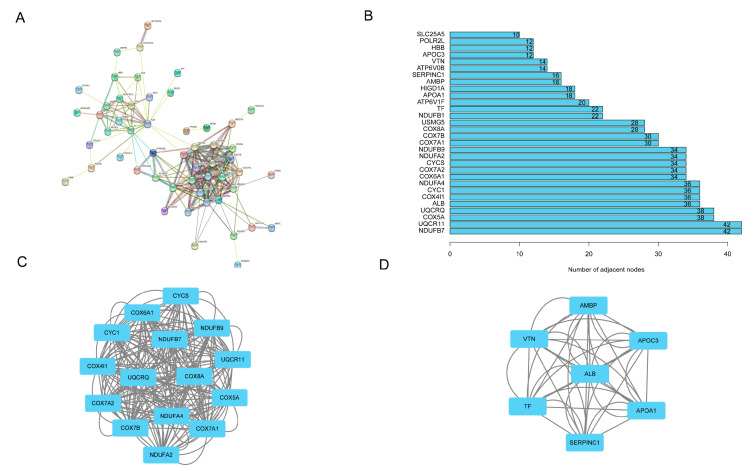
The PPI network and two submodules. (**A**) PPI network of the DEGs. (**B**) Stacked bar chart of top 30 hub genes. (**C**) PPI network of module 1. (**D**) PPI network of module 2.

We constructed a PPI network with 53 nodes and 442 edges using online tool STRING (Figure 9A) and visualized it in Cytoscape. The key genes with the top 30 node degrees in the network were listed in the bar chart (Figure 9B). Then, two significant modules containing 15 and 7 genes, respectively, were distinguished by MCODE (Figure 9C,D). The following GO and KEGG functional annotations revealed that the main functional areas of genes in submodule 1 were significantly enriched in oxidative phosphorylation, the aerobic electron transport chain, and ATP synthesis coupled electron transport. Genes in submodule 2 exhibited significant enrichment in the negative regulation of hydrolase activity, sterol import, and cholesterol import. The main pathways of genes in submodule 1 were enriched in oxidative phosphorylation, non-alcoholic fatty liver disease, and Parkinson’s disease. The genes in submodule 2 were enriched in cholesterol metabolism, the PPAR signaling pathway, and complement and coagulation cascades (Table 1 and Table 2).

### 3.6. HMCN1 Mutation-Related Tumor Immune Microenvironment

**Figure 10:**
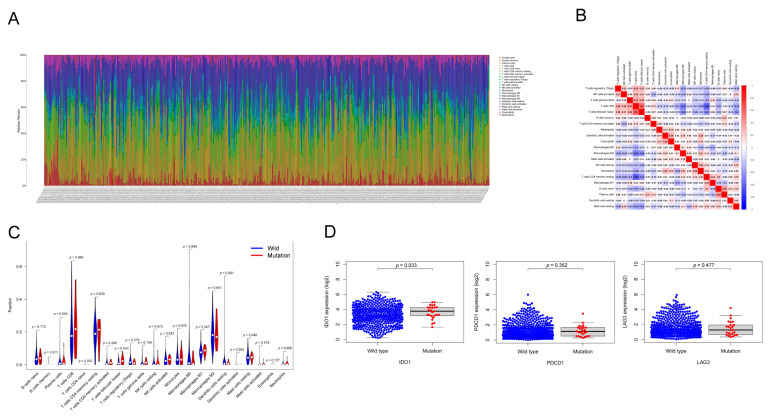
*HMCN1* mutation is related to tumor immune microenvironment. (**A**) The composition of 22 types of immune cells. (**B**) The correlation graph of immune cells. (**C**) The difference of immune cell proportion between mutant and wild-type samples. (**D**) Representative immune check point genes expression in mutant and wild-type samples.

The composition of 22 subsets of immune-infiltrating cells calculated via CIBERSORT was illustrated in the bar graph of Figure 10A. The relationship of different immune cell components was also demonstrated (Figure 10B). Then, we investigated the differences of immune cell distribution between different mutation status. The violin plot in Figure 10C presents that the abundance of T cells CD4 naïve and T cells follicular helper was significantly different in *HMCN1*-mutant samples. We also compared the expression of immune checkpoint genes between *HMCN1*-mutant and wild type samples, and revealed that in *HMCN1*-mutant samples, the expression of *IDO1* was significantly upregulated (Figure 10D).

## 4. Discussion

In the current study, we first reviewed the mutation profile of 537 American and 106 Asian ccRCC patients and identified three gene mutations that were related to both TMB and clinical outcome. Among them, the correlation of *HMCN1* mutation and ccRCC has not been reported yet. Further exploration revealed that in samples with *HMCN1* mutation, pathways related to metabolism were significantly enriched. Moreover, we observed a different infiltration level in naïve CD4 T and follicular helper T cells in the *HMCN1*-mutant group. The mutant samples also showed an increase in *IDO1* expression.

HMCN executes its biological functions as an extracellular matrix protein. The HMCN family has two orthologs (HMCN1 and 2). HMCN1 is predominantly generated by stromal cells [38]. The function of *HMCN1* has not been well explored yet. As a cell polarity-related gene commonly associated with calcium binding, the extracellular matrix component HMCN1 is found at the dermal–epidermal and tendon junctions and may be involved in the structural organization of epithelial cell junctions [38]. *HMCN1* has been confirmed to be related to AMD. Its variant Gln5345Arg has been discovered in a large AMD family in America [16], and in a small subgroup of AMD patients, it may contribute to disease susceptibility [39].

Mutations and the altered expression of *HMCN1* have been proved to be involved in malignant tumor development. *HMCN1* regulates cancer-associated fibroblasts (CAFs) to reinforce the aggressiveness of ovarian cancer. CAFs in high-grade plasmacytoid carcinoma and clear cell carcinoma tissues show an upregulation of *HMCN1*, and tumor cells tend to be less invasive after silencing *HMCN1* expression in fibroblasts [40]. In hepatocellular carcinoma, HMCN1 protein was also found to be overexpressed in tumors by proteomic analyses [41].

*HMCN1* mutation has been found in many cancers such as gastric, colorectal [42], prostate [43], triple-negative breast cancer [44], and small-cell gallbladder neuroendocrine carcinoma [45]. *HMCN1* mutation is associated with patients’ pathological characteristics and clinical prognoses. A study of molecular profiles and metastatic markers in Chinese gastric cancer patients indicated that samples carrying *HMCN1* mutation are associated with peritoneal metastasis [46]. The allelic mutation frequency of *HMCN1* is also significantly related to the prognosis of breast cancer [17]. As is known to all, drug resistance can be attributed to ECM by interfering with drug permeation into cancer tissues and inducing apoptosis resistance. Being an important component of ECM, *HMCN1* has a significantly lower expression in multi-drug resistant ovarian cancer cells [47].

In regards to kidney disease, *HMCN1* variants play a role in renal pathophysiology [48] and are considered as a potential gene causing diabetic nephropathy in Mexican Americans [49]. In addition, patients with proteinuric nephropathy have higher levels of *HMCN1* expression in their kidneys. In vitro and in vivo models reveal that *HMCN1* contributes to the remodeling of the podocyte cytoskeleton and the increased expression of *HMCN1* in podocytes can be stimulated in response to hyperglycemia [50], which is an important risk factor of RCC [51,52]. In fact, consistent with ovarian and liver cancers discussed above, we did observe a significant overexpression of *HMCN1* in ccRCC samples in TCGA cohort. However, the correlation between hyperglycemia, the expression of *HMCN1*, and the development of ccRCC is not yet clear.

Since *HMCN1* mutation plays an essential role in cancer development, it is necessary to elucidate the underlying mechanisms. Our GSEA results showed that *HMCN1* mutation was mainly associated with cellular metabolism-related pathways including glucose and lipid metabolism, which are extremely necessary for tumorigenesis and cancer progression [53].

Abnormal metabolism is an important hallmark of cancer. Tumors can gain growth advantages through metabolic reprogramming processes, such as aerobic glycolysis and increased lipid synthesis [54]. Previous studies have shown that the reprogramming of glucose and lipid metabolism occurs frequently in ccRCC [55]. Although conventional research tends to assume that tumor cells primarily undergo glycolysis without oxidative metabolism, recent studies have found that certain types of tumor cells consume more oxygen and have higher levels of oxidative phosphorylation than normal cells. The application of oxidative phosphorylation inhibitors may target at the procedure of tumor cell metabolism and thus exert anti-tumor efficacy [56]. The increase of oxidative phosphorylation and mitochondrial membrane fusion-mediated NADH/NAD+ metabolism can promote the immortalization of neural stem cell tumors [57]. Moreover, numerous pieces of research have shown that the electron transport chain in mitochondria is required for cancer development [58], which is consistent with the results of our GO functional annotation. There have also been studies demonstrating that there may be a crosstalk between energy metabolism and ECM remodeling, further supporting our assumption that *HMCN1* mutation might contribute to cellular metabolic reprogramming [59].

The present study found that key pathways altered in *HMCN1*-mutant ccRCC samples were oxidative phosphorylation and glucose and lipid metabolism, suggesting that drugs targeted at lipid and glucose metabolism and oxidative phosphorylation might contribute to developing new therapeutic strategies for ccRCC with *HMCN1* mutation.

In terms of immune cell infiltration conditions, naive CD4T and follicular helper T (Tfh) cells show significantly different abundance in *HMCN1* mutant samples, indicating that *HMCN1* mutation may enhance anti-tumor immune responses. Naïve CD4T cells differentiate towards Tfh cells in response to transcription factor B-cell lymphoma 6 [60,61]. Tfh cells belong to a special subgroup of CD4T cells, which are involved in the regulation of protective antibody responses to pathogens. The capabilities of mature Tfh cells are to assist B cells in promoting antibody affinity maturation, class switch reorganization, and memory cells production [62,63]. Besides, Tfh cells were most relevant to CD8T cells and exhibited the highest negative association with M2 macrophages, further confirming the hypothesis that an altered tumor immune microenvironment induced by *HMCN1* mutation may be involved in enhancing anti-tumor immunity.

Currently, an immune checkpoint inhibitor regimen shows impressive efficacy in multiple types of cancers [64,65,66]. Conventional immune checkpoints include *CTLA4*, *PDCD1*, *CD274*, *IDO1*, *LAG3*, *TIGIT* and so on [67]. *IDO1* is a rate-limiting metabolic enzyme in tryptophan metabolism, which can convert tryptophan into kynurenine [68]. Interferon α stimulates the expression of *IDO1* [69], which subsequently induces the inactivation of T cells and NK cells, and the promotion of Tregs and myeloid-derived suppressor cells (MDSCs) [70,71]. Numerous studies have shown that *IDO1* is significantly overexpressed in a variety of human cancers and mediates immunosuppression [72,73,74]. In the present study, we revealed that *HMCN1* mutation was associated with increased *IDO1* expression, indicating that *HMCN1* mutation may be a double-edged sword in regulating immune responses. Since our previous results have suggested that *HMCN1* mutation can affect metabolic pathways, it may be important to further explore the correlation between *HMCN1* mutation and anti-tumor immunity regulated by cellular metabolism.

Several limitations should be considered in this study. First, the sample size of TCGA dataset is limited and covers only patients from western countries, which makes it difficult to apply our conclusion to patients worldwide. There are also differences in age, gender, and ethnicity between samples, thus causing potential errors or biases. Because of insufficient expression and survival information in the UTokyo cohort, it is difficult for us to figure out whether *HMCN1* mutation is also a predictive biomarker of prognosis and the immune microenvironment in Japanese patients. Therefore, a further exploration of the *HMCN1* mutation pattern in Asian patients is necessary. Second, the TCGA dataset includes only 25 cases with *HMCN1* mutation, which might cause biased results. Finally, our present study is only a correlation study based on multidimensional data, which lacks relevant basic experiments in cell lines and clinical samples. In the future, we will carry out clinical and biological experiments to validate the function and clinical significance of *HMCN1* mutation in ccRCC and further explore the underlying mechanisms related to *HMCN1* mutation in cancers.

## 5. Conclusions

In summary, we provided detailed insights into the critical role of *HMCN1* mutation in regulating ccRCC progression. The present study suggested that *HMCN1* mutation occurs frequently in ccRCC and was related to a higher TMB and a poorer clinical outcome. *HMCN1* mutation was considered as an independent prognostic biomarker and may be relevant to cell metabolism and anti-tumor immunity.

## Figures and Tables

**Table 1:** Functional annotations of module 1.

ID	Description	*p*-Value	Genes
GO			
GO:0006119	Oxidative phosphorylation	6.61 × 10^−33^	*COX7B/COX8A/COX5A/CYC1/COX4I1/CYCS/NDUFB9/COX6A1/COX7A2/COX7A1/NDUFB7/UQCR11/NDUFA4/NDUFA2/UQCRQ*
GO:0019646	Aerobic electron transport chain	1.09 × 10^−32^	*COX8A/COX5A/CYC1/COX4I1/CYCS/NDUFB9/COX6A1/COX7A2/COX7A1/NDUFB7/UQCR11/NDUFA4/NDUFA2/UQCRQ*
GO:0042773	ATP synthesis coupled electron transport	4.11 × 10^−32^	*COX8A/COX5A/CYC1/COX4I1/CYCS/NDUFB9/COX6A1/COX7A2/COX7A1/NDUFB7/UQCR11/NDUFA4/NDUFA2/UQCRQ*
KEGG			
hsa00190	Oxidative phosphorylation	7.91 × 10^−28^	*COX7B/COX8A/COX5A/CYC1/COX4I1/CYCS/NDUFB9/COX6A1/COX7A2/COX7A1/NDUFB7/UQCR11/NDUFA4/NDUFA2/UQCRQ*
hsa04932	Non-alcoholic fatty liver disease	7.87 × 10^−27^	*COX7B/COX8A/COX5A/CYC1/COX4I1/CYCS/NDUFB9/COX6A1/COX7A2/COX7A1/NDUFB7/UQCR11/NDUFA4/NDUFA2/UQCRQ*
hsa05012	Parkinson disease	3.49 × 10^−23^	*COX7B/COX8A/COX5A/CYC1/COX4I1/CYCS/NDUFB9/COX6A1/COX7A2/COX7A1/NDUFB7/UQCR11/NDUFA4/NDUFA2/UQCRQ*

**Table 2:** Functional annotations of module 2.

ID	Description	*p*-Value	Genes
GO			
GO:0051346	Negative regulation of hydrolase activity	6.72 × 10^−8^	*VTN/SERPINC1/APOA1/APOC3/AMBP*
GO:0035376	Sterol import	5.38 × 10^−6^	*APOA1/APOC3*
GO:0070508	Cholesterol import	5.38 × 10^−6^	*APOA1/APOC3*
KEGG			
hsa04979	Cholesterol metabolism	0.00054577	*APOA1/APOC3*
hsa03320	PPAR signaling pathway	0.00122622	*APOA1/APOC3*
hsa04610	Complement and coagulation cascades	0.001572335	*VTN/SERPINC1*

## Data Availability

The public datasets analyzed in the current study are available in online repositories, including TCGA (http://portal.gdc.cancer.gov/projects (accessed on 13 February 2022)) and cBioportal (https://www.cbioportal.org/ (accessed on 13 February 2022)).

## References

[1] Linehan W.M., Ricketts C.J. (2019). The Cancer Genome Atlas of renal cell carcinoma: Findings and clinical implications. Nat. Rev. Urol..

[2] Deleuze A., Saout J., Dugay F., Peyronnet B., Mathieu R., Verhoest G., Bensalah K., Crouzet L., Laguerre B., Belaud-Rotureau M.A. (2020). Immunotherapy in Renal Cell Carcinoma: The Future Is Now. Int. J. Mol. Sci..

[3] Hsieh J.J., Purdue M.P., Signoretti S., Swanton C., Albiges L., Schmidinger M., Heng D.Y., Larkin J., Ficarra V. (2017). Renal cell carcinoma. Nat. Rev. Dis. Primers.

[4] Jonasch E., Gao J., Rathmell W.K. (2014). Renal cell carcinoma. BMJ.

[5] Adibi M., Thomas A.Z., Borregales L.D., Matin S.F., Wood C.G., Karam J.A. (2015). Surgical considerations for patients with metastatic renal cell carcinoma. Urol. Oncol..

[6] Eskicorapci S.Y., Teber D., Schulze M., Ates M., Stock C., Rassweiler J.J. (2007). Laparoscopic radical nephrectomy: The new gold standard surgical treatment for localized renal cell carcinoma. Sci. World J..

[7] Attalla K., Weng S., Voss M.H., Hakimi A.A. (2020). Epidemiology, Risk Assessment, and Biomarkers for Patients with Advanced Renal Cell Carcinoma. Urol. Clin. N. Am..

[8] Motzer R.J., Russo P., Nanus D.M., Berg W.J. (1997). Renal cell carcinoma. Curr. Probl. Cancer.

[9] Itsumi M., Tatsugami K. (2010). Immunotherapy for renal cell carcinoma. Clin. Dev. Immunol..

[10] Albiges L., Powles T., Staehler M., Bensalah K., Giles R.H., Hora M., Kuczyk M.A., Lam T.B., Ljungberg B., Marconi L. (2019). Updated European Association of Urology Guidelines on Renal Cell Carcinoma: Immune Checkpoint Inhibition Is the New Backbone in First-line Treatment of Metastatic Clear-cell Renal Cell Carcinoma. Eur. Urol..

[11] Chan T.A., Yarchoan M., Jaffee E., Swanton C., Quezada S.A., Stenzinger A., Peters S. (2019). Development of tumor mutation burden as an immunotherapy biomarker: Utility for the oncology clinic. Ann. Oncol..

[12] Jardim D.L., Goodman A., de Melo Gagliato D., Kurzrock R. (2021). The Challenges of Tumor Mutational Burden as an Immunotherapy Biomarker. Cancer cell.

[13] Qiao M., Jiang T., Liu X., Mao S., Zhou F., Li X., Zhao C., Chen X., Su C., Ren S. (2021). Immune Checkpoint Inhibitors in EGFR-Mutated NSCLC: Dusk or Dawn?. J. Thorac. Oncol..

[14] Baba Y., Nomoto D., Okadome K., Ishimoto T., Iwatsuki M., Miyamoto Y., Yoshida N., Baba H. (2020). Tumor immune microenvironment and immune checkpoint inhibitors in esophageal squamous cell carcinoma. Cancer Sci..

[15] Lin M.H., Pope B.D., Sasaki T., Keeley D.P., Sherwood D.R., Miner J.H. (2020). Mammalian hemicentin 1 is assembled into tracks in the extracellular matrix of multiple tissues. Dev. Dyn..

[16] Schultz D.W., Klein M.L., Humpert A.J., Luzier C.W., Persun V., Schain M., Mahan A., Runckel C., Cassera M., Vittal V. (2003). Analysis of the ARMD1 locus: Evidence that a mutation in HEMICENTIN-1 is associated with age-related macular degeneration in a large family. Hum. Mol. Genet..

[17] Kikutake C., Yoshihara M., Sato T., Saito D., Suyama M. (2018). Intratumor heterogeneity of HMCN1 mutant alleles associated with poor prognosis in patients with breast cancer. Oncotarget.

[18] The Cancer Genome Atlas. http://portal.gdc.cancer.gov/projects.

[19] Sato Y., Yoshizato T., Shiraishi Y., Maekawa S., Okuno Y., Kamura T., Shimamura T., Sato-Otsubo A., Nagae G., Suzuki H. (2013). Integrated molecular analysis of clear-cell renal cell carcinoma. Nat. Genet..

[20] cBio Cancer Genomics Portal. https://www.cbioportal.org/.

[21] Skidmore Z.L., Campbell K.M., Cotto K.C., Griffith M., Griffith O.L. (2021). Exploring the Genomic Landscape of Cancer Patient Cohorts with GenVisR. Curr. Protoc..

[22] Chalmers Z.R., Connelly C.F., Fabrizio D., Gay L., Ali S.M., Ennis R., Schrock A., Campbell B., Shlien A., Chmielecki J. (2017). Analysis of 100,000 human cancer genomes reveals the landscape of tumor mutational burden. Genome Med..

[23] Yin W., Jiang X., Tan J., Xin Z., Zhou Q., Zhan C., Fu X., Wu Z., Guo Y., Jiang Z. (2020). Development and Validation of a Tumor Mutation Burden-Related Immune Prognostic Model for Lower-Grade Glioma. Front. Oncol..

[24] Parikh K., Huether R., White K., Hoskinson D., Beaubier N., Dong H., Adjei A.A., Mansfield A.S. (2020). Tumor Mutational Burden From Tumor-Only Sequencing Compared With Germline Subtraction From Paired Tumor and Normal Specimens. JAMA Netw. Open.

[25] Robinson M.D., McCarthy D.J., Smyth G.K. (2010). edgeR: A Bioconductor package for differential expression analysis of digital gene expression data. Bioinformatics.

[26] Yu G., Wang L.G., Han Y., He Q.Y. (2012). clusterProfiler: An R package for comparing biological themes among gene clusters. OMICS J. Integr. Biol..

[27] Subramanian A., Tamayo P., Mootha V.K., Mukherjee S., Ebert B.L., Gillette M.A., Paulovich A., Pomeroy S.L., Golub T.R., Lander E.S. (2005). Gene set enrichment analysis: A knowledge-based approach for interpreting genome-wide expression profiles. Proc. Natl. Acad. Sci. USA.

[28] Liberzon A., Birger C., Thorvaldsdóttir H., Ghandi M., Mesirov J.P., Tamayo P. (2015). The Molecular Signatures Database (MSigDB) hallmark gene set collection. Cell Syst..

[29] von Mering C., Huynen M., Jaeggi D., Schmidt S., Bork P., Snel B. (2003). STRING: A database of predicted functional associations between proteins. Nucleic Acids Res..

[30] Smoot M.E., Ono K., Ruscheinski J., Wang P.L., Ideker T. (2011). Cytoscape 2.8: New features for data integration and network visualization. Bioinformatics.

[31] Rhrissorrakrai K., Gunsalus K.C. (2011). MINE: Module Identification in Networks. BMC Bioinform..

[32] Newman A.M., Liu C.L., Green M.R., Gentles A.J., Feng W., Xu Y., Hoang C.D., Diehn M., Alizadeh A.A. (2015). Robust enumeration of cell subsets from tissue expression profiles. Nat. Methods.

[33] Ritchie M.E., Phipson B., Wu D., Hu Y., Law C.W., Shi W., Smyth G.K. (2015). limma powers differential expression analyses for RNA-sequencing and microarray studies. Nucleic Acids Res..

[34] Hu K. (2020). Become Competent within One Day in Generating Boxplots and Violin Plots for a Novice without Prior R Experience. Methods Protoc..

[35] Peña-Llopis S., Vega-Rubín-de-Celis S., Liao A., Leng N., Pavía-Jiménez A., Wang S., Yamasaki T., Zhrebker L., Sivanand S., Spence P. (2012). BAP1 loss defines a new class of renal cell carcinoma. Nat. Genet..

[36] Jin S., Wu J., Zhu Y., Gu W., Wan F., Xiao W., Dai B., Zhang H., Shi G., Shen Y. (2018). Comprehensive Analysis of BAP1 Somatic Mutation in Clear Cell Renal Cell Carcinoma to Explore Potential Mechanisms in Silico. J. Cancer.

[37] Li P., Xiao J., Zhou B., Wei J., Luo J., Chen W. (2020). SYNE1 mutation may enhance the response to immune checkpoint blockade therapy in clear cell renal cell carcinoma patients. Aging.

[38] Welcker D., Stein C., Feitosa N.M., Armistead J., Zhang J.L., Lütke S., Kleinridders A., Brüning J.C., Eming S.A., Sengle G. (2021). Hemicentin-1 is an essential extracellular matrix component of the dermal-epidermal and myotendinous junctions. Sci. Rep..

[39] Fisher S.A., Rivera A., Fritsche L.G., Keilhauer C.N., Lichtner P., Meitinger T., Rudolph G., Weber B.H. (2007). Case-control genetic association study of fibulin-6 (FBLN6 or HMCN1) variants in age-related macular degeneration (AMD). Hum. Mutat..

[40] Liu C.L., Pan H.W., Torng P.L., Fan M.H., Mao T.L. (2019). SRPX and HMCN1 regulate cancer-associated fibroblasts to promote the invasiveness of ovarian carcinoma. Oncol. Rep..

[41] Elmas A., Lujambio A., Huang K.L. (2022). Proteomic Analyses Identify Therapeutic Targets in Hepatocellular Carcinoma. Front. Oncol..

[42] Lee S.H., Je E.M., Yoo N.J., Lee S.H. (2015). HMCN1, a cell polarity-related gene, is somatically mutated in gastric and colorectal cancers. Pathol. Oncol. Res..

[43] Zhao X., Lei Y., Li G., Cheng Y., Yang H., Xie L., Long H., Jiang R. (2019). Integrative analysis of cancer driver genes in prostate adenocarcinoma. Mol. Med. Rep..

[44] Saravia C.H., Flores C., Schwarz L.J., Bravo L., Zavaleta J., Araujo J., Neciosup S., Pinto J.A. (2019). Patterns of Mutation Enrichment in Metastatic Triple-Negative Breast Cancer. Clin. Med. Insights Oncol..

[45] Li M., Liu F., Zhang Y., Wu X., Wu W., Wang X.A., Zhao S., Liu S., Liang H., Zhang F. (2017). Whole-genome sequencing reveals the mutational landscape of metastatic small-cell gallbladder neuroendocrine carcinoma (GB-SCNEC). Cancer Lett..

[46] Chen C., Shi C., Huang X., Zheng J., Zhu Z., Li Q., Qiu S., Huang Z., Zhuang Z., Wu R. (2019). Molecular Profiles and Metastasis Markers in Chinese Patients with Gastric Carcinoma. Sci. Rep..

[47] Januchowski R., Zawierucha P., Ruciński M., Zabel M. (2014). Microarray-based detection and expression analysis of extracellular matrix proteins in drug-resistant ovarian cancer cell lines. Oncol. Rep..

[48] Thompson C.L., Klein B.E., Klein R., Xu Z., Capriotti J., Joshi T., Leontiev D., Lee K.E., Elston R.C., Iyengar S.K. (2007). Complement factor H and hemicentin-1 in age-related macular degeneration and renal phenotypes. Hum. Mol. Genet..

[49] Kim S., Abboud H.E., Pahl M.V., Tayek J., Snyder S., Tamkin J., Alcorn H., Ipp E., Nast C.C., Elston R.C. (2010). Examination of association with candidate genes for diabetic nephropathy in a Mexican American population. Clin. J. Am. Soc. Nephrol. CJASN.

[50] Toffoli B., Zennaro C., Winkler C., Giordano Attianese G.M.P., Bernardi S., Carraro M., Gilardi F., Desvergne B. (2018). Hemicentin 1 influences podocyte dynamic changes in glomerular diseases. Am. J. Physiol. Renal. Physiol..

[51] Labochka D., Moszczuk B., Kukwa W., Szczylik C., Czarnecka A.M. (2016). Mechanisms through which diabetes mellitus influences renal cell carcinoma development and treatment: A review of the literature. Int. J. Mol. Med..

[52] Lee H., Kwak C., Kim H.H., Byun S.S., Lee S.E., Hong S.K. (2015). Diabetes Mellitus as an Independent Predictor of Survival of Patients Surgically Treated for Renal Cell Carcinoma: A Propensity Score Matching Study. J. Urol..

[53] DeBerardinis R.J., Chandel N.S. (2016). Fundamentals of cancer metabolism. Sci. Adv..

[54] Park J.H., Pyun W.Y., Park H.W. (2020). Cancer Metabolism: Phenotype, Signaling and Therapeutic Targets. Cells.

[55] Wettersten H.I., Aboud O.A., Lara P.N., Weiss R.H. (2017). Metabolic reprogramming in clear cell renal cell carcinoma. Nat. Rev. Nephrol..

[56] Ashton T.M., McKenna W.G., Kunz-Schughart L.A., Higgins G.S. (2018). Oxidative Phosphorylation as an Emerging Target in Cancer Therapy. Clin. Cancer Res. Off. J. Am. Assoc. Cancer Res..

[57] Bonnay F., Veloso A., Steinmann V., Köcher T., Abdusselamoglu M.D., Bajaj S., Rivelles E., Landskron L., Esterbauer H., Zinzen R.P. (2020). Oxidative Metabolism Drives Immortalization of Neural Stem Cells during Tumorigenesis. Cell.

[58] Martínez-Reyes I., Cardona L.R., Kong H., Vasan K., McElroy G.S., Werner M., Kihshen H., Reczek C.R., Weinberg S.E., Gao P. (2020). Mitochondrial ubiquinol oxidation is necessary for tumour growth. Nature.

[59] Romani P., Valcarcel-Jimenez L., Frezza C., Dupont S. (2021). Crosstalk between mechanotransduction and metabolism. Nat. Rev. Mol. Cell Biol..

[60] Johnston R.J., Poholek A.C., DiToro D., Yusuf I., Eto D., Barnett B., Dent A.L., Craft J., Crotty S. (2009). Bcl6 and Blimp-1 are reciprocal and antagonistic regulators of T follicular helper cell differentiation. Science.

[61] Nurieva R.I., Chung Y., Martinez G.J., Yang X.O., Tanaka S., Matskevitch T.D., Wang Y.H., Dong C. (2009). Bcl6 mediates the development of T follicular helper cells. Science.

[62] Song W., Craft J. (2019). T follicular helper cell heterogeneity: Time, space, and function. Immunol. Rev..

[63] Crotty S. (2014). T follicular helper cell differentiation, function, and roles in disease. Immunity.

[64] Hodi F.S., O’Day S.J., McDermott D.F., Weber R.W., Sosman J.A., Haanen J.B., Gonzalez R., Robert C., Schadendorf D., Hassel J.C. (2010). Improved survival with ipilimumab in patients with metastatic melanoma. N. Engl. J. Med..

[65] Borghaei H., Paz-Ares L., Horn L., Spigel D.R., Steins M., Ready N.E., Chow L.Q., Vokes E.E., Felip E., Holgado E. (2015). Nivolumab versus Docetaxel in Advanced Nonsquamous Non-Small-Cell Lung Cancer. N. Engl. J. Med..

[66] Garon E.B., Rizvi N.A., Hui R., Leighl N., Balmanoukian A.S., Eder J.P., Patnaik A., Aggarwal C., Gubens M., Horn L. (2015). Pembrolizumab for the treatment of non-small-cell lung cancer. N. Engl. J. Med..

[67] Darvin P., Toor S.M., Sasidharan Nair V., Elkord E. (2018). Immune checkpoint inhibitors: Recent progress and potential biomarkers. Exp. Mol. Med..

[68] Zhai L., Ladomersky E., Lenzen A., Nguyen B., Patel R., Lauing K.L., Wu M., Wainwright D.A. (2018). IDO1 in cancer: A Gemini of immune checkpoints. Cell. Mol. Immunol..

[69] Werner-Felmayer G., Werner E.R., Fuchs D., Hausen A., Reibnegger G., Wachter H. (1989). Characteristics of interferon induced tryptophan metabolism in human cells in vitro. Biochim. Biophys. Acta.

[70] Jitschin R., Braun M., Büttner M., Dettmer-Wilde K., Bricks J., Berger J., Eckart M.J., Krause S.W., Oefner P.J., Le Blanc K. (2014). CLL-cells induce IDOhi CD14+HLA-DRlo myeloid-derived suppressor cells that inhibit T-cell responses and promote TRegs. Blood.

[71] Yu J., Du W., Yan F., Wang Y., Li H., Cao S., Yu W., Shen C., Liu J., Ren X. (2013). Myeloid-derived suppressor cells suppress antitumor immune responses through IDO expression and correlate with lymph node metastasis in patients with breast cancer. J. Immunol..

[72] Tang K., Wu Y.H., Song Y., Yu B. (2021). Indoleamine 2,3-dioxygenase 1 (IDO1) inhibitors in clinical trials for cancer immunotherapy. J. Hematol. Oncol..

[73] Munn D.H., Mellor A.L. (2013). Indoleamine 2,3 dioxygenase and metabolic control of immune responses. Trends Immunol..

[74] Ball H.J., Sanchez-Perez A., Weiser S., Austin C.J., Astelbauer F., Miu J., McQuillan J.A., Stocker R., Jermiin L.S., Hunt N.H. (2007). Characterization of an indoleamine 2,3-dioxygenase-like protein found in humans and mice. Gene.

